# The Effects of Brain Tumours upon Medical Decision-Making Capacity

**DOI:** 10.1007/s11912-019-0793-3

**Published:** 2019-05-02

**Authors:** Will Hewins, Karolis Zienius, James L. Rogers, Simon Kerrigan, Mark Bernstein, Robin Grant

**Affiliations:** 1 Department of Clinical Neurosciences, Western General Hospital, Edinburgh, EH4 2XU Scotland; 2 Centre for Clinical Brain Sciences, University of Edinburgh, Edinburgh, EH16 4SB UK; 3 Tulane University, New Orleans, LA USA; 4 Department of Neurology, Salford Royal NHS Foundation Trust, Stott Lane, Salford, M6 8HD UK; 5 Division of Neurosurgery, Toronto Western Hospital, University of Toronto, Toronto, Canada

**Keywords:** Brain tumour, Capacity, Shared decision-making, Legal consent, Glioma, Brain metastasis

## Abstract

**Purpose of Review:**

Informed consent is the integral part of good medical practice in patients with brain tumours. Capacity to consent may be affected by the brain disorder or its treatment. We intend to draw upon the current neuro-oncology literature to discuss the influence intracranial tumours have upon patients’ capacity to consent to treatment and research.

**Recent Findings:**

We performed a systematic review of studies of capacity to consent for treatment or research in patients with intracranial tumours. The search retrieved 1597 papers of which 8 were considered eligible for review.

**Summary:**

Although there are obvious inherent limitations to solely assessing cognition, most research consistently demonstrated increased risk of incapacity in brain tumour patients with cognitive impairment. Specific items in cognitive screening batteries, for example Semantic Verbal Fluency Test (SVFT), Hopkins Verbal Learning Test (HVLT-Recall), and Trail Making Test A/B (TMT), are simple, easily applied tests that may act as significant red flags to identify patients at increased risk of incapacity and who subsequently will require additional cognitive/psychiatric evaluation or more formal tests for capacity to consent for treatment or research.

## Introduction

Before surgical treatment can take place, informed consent must be obtained from the adult patient. Informed consent requires that the patient be an adult and that they receive all relevant information, in an appropriate format, to enable the patient to understand, remember, evaluate, and communicate their decision. The patient must possess the prerequisite mental capacity to come to an autonomous and informed decision about their care. All adults are assumed to possess the mental capacity to make medical decisions about their care. Only at the point an individual demonstrates a deficit in cognition or disturbed mental state should assessment of their mental capacity be initiated. At this point, it is the treating physician’s duty of care to identify and attempt to accommodate the patient’s ability to understand and retain the relevant information long enough for them to weigh-up and communicate their decision. The effects of any underlying condition or side effects of prior treatment must also be considered. A myriad of complications from infection to general confusion can cause temporary loss of capacity. In cases where it is possible to wait for these factors to be treated or overcome, such steps should be taken before obtaining legal consent for surgical procedures. After all appropriate support has been exhausted, if the patient still cannot make a reasoned decision regarding the procedure, the patient is considered to have incapacity. The assessment of incapacity must always remain specific to both the patient and their given treatment decision. Judgement of incapacity can change throughout a patient’s illness and should be seen as a continually evolving set of constructs (see Mental Capacity Act, 2005 [1]) that may affect medical decision-making.

Whilst capacity must be assessed on a case-by-case basis, certain risk factors [2, 3] and medical conditions are associated with an increased likelihood of incapacity [4, 5]. The largest body of literature comes from neurodegenerative and psychiatric conditions [6–8], in which disturbance of thought or cognition is often noted alongside incapacity [9]. Patients with intra-cerebral tumours often present with cognitive impairment from the tumour itself or treatment side effects [10] and are at a high risk of having incapacity [11].

We have performed a systematic review of studies of capacity to consent for treatment or research in patients with brain tumours.

## Methods

### Literature Searches

A preliminary scoping search (Appendix 1) was conducted to classify study identifiers to be used in the full systematic review. The scoping search drew 1133 research articles which were screened by a researcher. The findings from this search were only used in identifying appropriate parameters for a detailed literature search and no data were extracted.

The full literature search strategy (Appendix 2) was developed in conjunction with an information specialist at Cochrane Neuro-Oncology. The full Embase directory was used when applying the search strategy yielding 1596 results.

### Study Eligibility

**Fig. 1:**
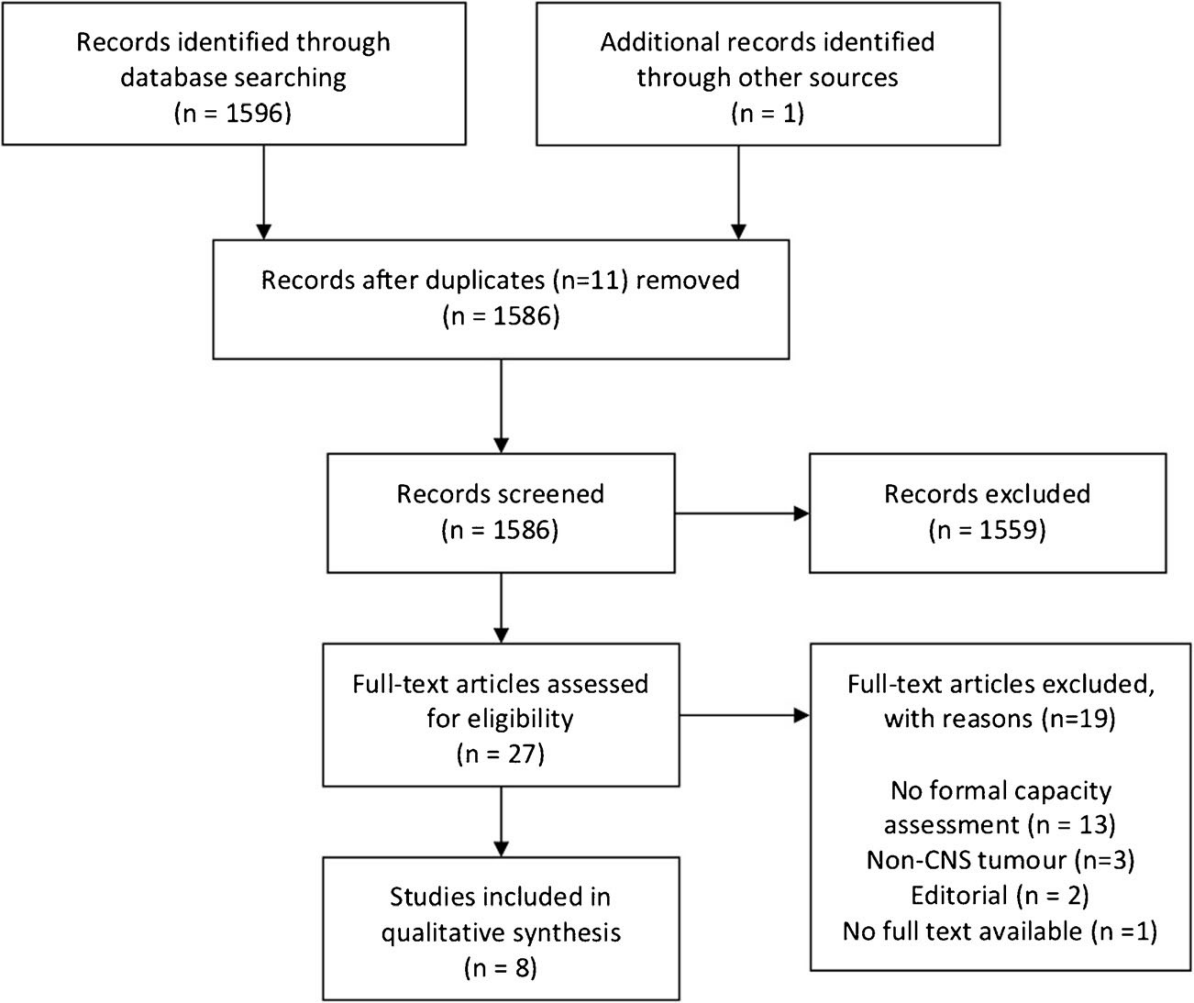
Adapted PRISMA flow diagram [12]

After removal of duplicate articles, titles and abstracts of 1586 research articles were initially screened for immediate inclusion criteria. Due to the relatively limited findings of the scoping search, only two standards were used to identify papers suited for further assessment: (1) patient population were adults aged 16 years or older who were diagnosed with a central nervous system tumour and (2) capacity to consent to either research or medical treatment as an outcome measure. Twenty-seven research articles were identified as eligible for full-text assessment. Upon full-text assessment, 19 of these papers were deemed ineligible (see Fig. 1). Eight full-text articles were included in the present review. A brief summary of the six studies we extracted data from is shown in Table 1. Due to the limited results and variance in methodologies utilised by researchers, quantitative analyses were not performed.

**Table 1:** A brief description of studies and their main findings used in the present review

Study	Diagnosis	Number of patients	Control	Capacity assessment	Test timepoint (consent)	Main findings
Triebel et al. [18]	Malignant glioma	26	Yes	CCTI	Average time from diagnosis = 6.9 months	Over 50% of patients showed compromised capacity in medical decision-making. Cognitive performance on verbal acquisition and recall, in addition to semantic fluency, predicted performance of the appreciation, reasoning, and understanding standards of consent.
Marson et al. [30]	Malignant glioma	26	Yes	CCRI	Average time from diagnosis = 6.9 months	Malignant glioma patients performed significantly below the controls on the consent standards of appreciation, reasoning, and understanding. Around one-third of patients showed compromised capacity. Phonemic and semantic verbal fluency found to predict CCRI performance.
Kerrigan et al. [29]	Radiologically suspected intracranial tumour	100	No	MacCAT-T	Preoperative (no consent required)	25% of patients lacked mental capacity to give valid consent to neurosurgery, of which almost half were missed on initial capacity assessment by the neurosurgical team. Patients lacking mental capacity were significantly more cognitively impaired than those with capacity. ACE-R semantic verbal fluency performance and ability to repeat 7-item name and address after three attempts were predictive of incapacity.
Gerstenecker et al. [31••]	Brain metastasis	41	Yes	CCTI	Within a week before starting RT	The *understanding* facet of capacity was associated with a range of cognitive performances. Also, performance in phonemic fluency and verbal memory were found to be predictors of capacity to understand a treatment decision.
Gerstenecker et al. [32••]	Brain metastasis	41	Yes	CCTI	Within a week before starting RT	The *reasoning* facet of capacity shared significant associations with two cognitive performance aspects. Also, episodic memory and processing speed performance were found to be predictive of capacity to reason through a treatment decision.

*Patients with a diagnosis of either a primary or metastatic brain tumour were included in the study

Requirements—direct assessment of capacity using either MACCAT-T or CCTR/CCRI

*ACE-R*, Addenbrooke’s Cognitive Examination-revised; *CCRI*, Capacity to Consent to Research Instrument; *CCTI*, Capacity to Consent to Treatment Instrument; *KPS*, Karnofsky Performance Scale; *MACCAT-T*, MacArthur Competence Assessment Tool for Treatment

## The Assessment of Mental Capacity

Whilst there is not yet a gold-standard assessment of capacity, several tools exist to aid assessment [13–15]. Three such assessments are the MacArthur Competence Assessment Tool for Treatment (MacCAT-T), Competency to Consent to Treatment Instrument (CCTI), and the Competency to Consent to Research Instrument (CCRI). The MacCAT-T offers clinicians a semi-structured interview process to ensure all facets of capacity are discussed and appraised before assessment is made. The MacCAT-T utilises the real-world proposed treatment option with the patient and has seen good clinical utility in general inpatient, psychiatric, and end-of-life cancer populations [2, 6, 16]. The CCTI and CCRI on the other hand offer hypothetical treatment or research vignettes to which a patient is assessed on their performance across the four constructs of capacity (understanding, appreciation, reasoning, and expression of choice). Whilst the instruments have seen utility across a range of populations [17, 18] and all three assessments are deemed substantially more robust than clinician appraisal alone, they all still rely on the clinician’s subjective appraisal of information portrayed by the patient.

### Cognitive Assessment

Theorised cognitive underpinnings of capacity [19, 20] have been compared to standardised cognitive batteries in an attempt to simplify and standardise the binary decision whether a patient has or does not have capacity to make a decision around a specific treatment, such as, surgery [21]. Immediate limitations to using such methods revolve around the duration of administering detailed neuropsychological assessments. Instead of in-depth, and often arduous neuropsychological assessments, abbreviated cognitive batteries have been considered as potential stand-ins for capacity assessment [22, 23]. The poor sensitivity of the Mini-Mental State Examination (MMSE) alone limits its use as a formal capacity assessment [24]. Further, the MMSE lacks sensitivity to executive functions [25, 26], a domain regularly shown to be important when assessing decision-making and mental capacity [27, 28].

Updated approaches to the rapid assessment of cognitive status such as the Addenbrooke’s Cognitive Examination (ACE) and the Montreal Cognitive Assessment (MoCA) are both considered more sensitive than the MMSE and, amongst other shared neurocognitive subtests, both include brief assessment of executive function. Importantly, the ACE has been demonstrated as a sensitive test for patients with brain tumour, when screening for capacity to give consent prior to surgery compared with the MacCAT-T, performed by a dual-trained lawyer and neurologist [29]. In this study, investigating capacity in suspected intracranial tumour patients found that 25% of patients were found to lack the mental capacity required to consent to their neurosurgical treatment. Of particular note, the authors report poor performance on the semantic verbal fluency subtest (SVFT—‘how many animals can you think of in a minute’) to predict incapacity with 96% sensitivity and 63% specificity. The addition of a brief cognitive test involving the recall of a 7-item name and address after three attempts to memorise it increased the sensitivity to 100%, specificity 83%, and 66% positive predictive value compared with the MacCAT-T. These subtests, however, should not be seen as a proxy for capacity assessment [22, 29]. Rather, poor performance on cognitive assessment should be considered as a ‘red flag’, mandating a more rigorous capacity assessment.

## Mental Capacity in Brain Tumour

The systematic review identified two case control studies assessing capacity in known primary brain tumour (PBT) patients following surgery. Patients and controls were consented, and capacity was assessed using a hypothetical scenario for treatment. Triebel and colleagues [18] found that compared to controls, PBT patients were impaired using the CCTI capacity constructs of reasoning and understanding (35% and 54%, respectively) and 23% of patients had a compromised construct of appreciation that approached statistical significance (*p* = 0.06). Cognitive performance in tests of verbal memory, semantic fluency, and executive function showed strongest associations with appreciation. Multivariate analysis indicated around half of appreciation performance variance was attributable to Hopkins Verbal Learning Test (HVLT) recognition discrimination index and animal word fluency test performance. Both reasoning and understanding showed strongest associations with verbal acquisition and recall (HVLT total), semantic fluency (animal word fluency), and executive function (Trail Making Test B). Multivariate analysis indicated Total HVLT performance to account for 33% of patient variance in reasoning, whereas combined HVLT total and animal word fluency performance accounted for 72% of the variance in understanding scores.

When assessing research consent capacity using the CCRI, understanding appeared once again most commonly impaired [30]. Unlike treatment consent, more patients were impaired in appreciation than reasoning (31% and 23%, respectively). Multivariate analyses suggested semantic word fluency as the sole predictor for the appreciation standard of the CCRI, accounting for 61% of its variance. Phonemic fluency accounted for 30% variance in reasoning performance and both phonemic fluency and semantic fluency emerged as the two-step multivariate predictors of understanding, accounting for 71% of the variance. Again, no such associations were drawn between any cognitive performance and the consent facet of expressing a choice.

There are clear similarities between the two studies investigating primary brain tumours and capacity [18, 30]. Much like the aforementioned findings of Kerrigan [29] where semantic verbal fluency score ≤ 10 words in a minute was associated with lack of capacity to consent for surgery, verbal memory and verbal fluency offered potential ‘red flags’ whereby poor performance could indicate the need to further assess capacity.

Three further studies were identified reporting on capacity in cerebral metastases [31••, 32••, 33•]. Triebel and colleagues report 61% of all patients diagnosed with brain metastases showed some form of deficit in capacity (< 1.5 SD below the mean in one construct area) when assessed within a week of starting radiotherapy for their brain metastases [33•]. Only one patient showed impaired expression of treatment choice, with a further 17% of patients showing impairment in appreciation, 39% in reasoning, and 46% of patients showing some form of impairment in their ability to understand treatment decisions. This apparent rank order of incidence likely relates to the order of complexity each facet assesses [34, 35]. Significant differences between control and patient performance were only reported in reasoning and understanding.

In the pair of papers presented by Gerstenecker and colleagues, 41 patients with diagnosed brain metastases were compared against demographically matched controls for cognitive predictors of understanding [31••] and reasoning through [32••] treatment decisions. Forty-six percent of patients were found to have impaired understanding, as defined by a score of ≥ 1.5 standard deviation below control group average performance. By the same definition, 39% of patients were found to present impaired decision-making capacity in reasoning through a treatment decision. The capacity construct of reasoning was also significantly associated with cognitive performance in verbal memory and processing speed. As such, the researchers were able to report an equation to predict the likelihood of a patient presenting with impaired or intact reasoning capacity utilising test scores on both HVLT Delayed Recall and Trail Making Test A [32••]. Similar associations were also reported in Gerstenecker et al.’s paper investigating the capacity construct of understanding [31••]. As was seen in the reasoning construct, both delayed recall and trail making tests were significantly associated with understanding. In addition to these, several other cognitive performances were found significant (see Table 2). Of note, performance in phonemic fluency and HVLT total score allowed logistic regression predictions to impaired or intact understanding to be performed.

**Table 2:** A summary of statistically significant regression analyses as reported in reviewed research

Capacity function	Author (year)	Patient sample	Statistical analysis	Cognitive test	Statistic/significance
Appreciation	Triebel et al. [18]	Malignant glioma	Stepwise regression	HVLT-RDI Animal fluency	*R*^2^ = 0.50*** R^2^ = 0.58*
	Marson et al. [30]	Malignant glioma	Stepwise regression	Animal fluency	*R*^2^ = 0.62***
Reasoning	Triebal et al. [18]	Malignant glioma	Stepwise regression	HVLT trials 1–3	*R*^2^ = 0.36***
	Marson et al. [30]	Malignant glioma	Stepwise regression	Letter fluency	*R*^2^ = 0.34**
	Gerstenecker et al. [32••]	Brain metastases	Linear regression	HVLT delayed and TMT A	*R*^2^ = 0.18*
Understanding	Triebal et al. [18]	Malignant glioma	Stepwise regression	HVLT trials 1–3 Animal fluency Trial B	*R*^2^ = 0.68*** R^2^ = 0.75* r = − 0.79**
	Marson et al. [30]	Malignant glioma	Stepwise regression	Letter fluency Animal fluency	*R*^2^ = 0.64*** R^2^ = 0.73***
	Gerstenecker et al. [31••]	Brain metastases	Stepwise regression	HVLT total HVLT total and phonemic fluency	*R*^2^ = 0.58*** R^2^ = 0.68***

Significance levels: *p ≤ .05, **p ≤ .01, ***p ≤ .001

All *R*^2^ reported are cumulative, not adjusted

## The Effect of Tumour and Patient Profiles

### Tumour Location

No studies identified in the present literature review specifically investigated the effects of tumour location on capacity. There is, however, an opportunity to draw indirect associations from a growing body of literature regarding lesion sites and their associated cognitive impairment [36, 37]. Mattavelli and colleagues, for example, report a deficit in cognitive decision-making in frontal low-grade glioma [38]. Whilst pragmatically similar, poor performance in a cognitive decision-making task cannot be translated to poor medical decision-making without formal capacity assessment. Due to theorised higher order cognitive underpinnings of capacity [20, 29, 39, 40], constructs of capacity are unlikely attributable to any one specific neural location nor tumour site. Instead, it is much more conceivable that damage to any number of locations associated with that cognitive function and in turn capacity performance to manifest in impairment.

### Tumour Grade

Patients with low-grade glioma most commonly present with seizures, whilst those with high-grade glioma more commonly present with focal neurological or cognitive deficits and headache associated with raised intracranial pressure. More recently, tumour grade has been considered more important with respect to neurocognitive abilities than tumour volume, seizure status, or concomitant medication in newly diagnosed glioma [41, 42]. Kerrigan and colleagues reported the incidence of incapacity in relation to WHO tumour grade [11, 29]. Lack of capacity to consent was associated with glioblastoma suggesting that the rapid tumour growth may affect neurocognitive performance by limiting the extent neuro-plasticity can accommodate lesion-based changes in the brain [36, 43]. Importantly, cognitive functions found by Noll et al. to be influenced by tumour grade were in line with those associated with capacity, specifically, verbal learning, executive function, and language ability [41]. Such associations specifically in the context of capacity assessment warrant further investigation with the intent of uncovering additional risk factors to the cognitive red flags described earlier.

### Effects of Treatment

The effect of surgery on a patient’s capacity to consent to subsequent treatments is likely an area of substantial interest due to the potential of side effects and relatively short interlude between follow-up treatments [37]. With respect to subsequent treatments, only one study reported the frequency of incapacity in relation to their treatment [32••]. No significant differences were seen between radiation and chemotherapy and no correction was made to account for patients who had both treatments. The limited data currently available leave this question open for further study. It is likely that both treatments have at least a transient effect on cognition [44] with the impact and duration of side effects often varying between treatment approaches [45–47], their influence on capacity may also be transient in nature. There are, however, increasing concerns over the persistence of cognitive impairment experienced following whole brain radiation therapy; as such, treatment fields and dose should be considered when making capacity assessment. There is growing evidence that hippocampal avoidance techniques may limit future cognitive impairment [48–50] and such techniques should be considered in reference to treatment influence on capacity.

Only one study reviewed reported on associations between current medication use and mental capacity. Marson et al. (2010) found that corticosteroid use was significantly associated with impaired capacity for appreciation, reasoning, and understanding [30]. Anticonvulsant medication was also associated with reasoning and understanding, although to a lesser extent. From the data presented by Marson and colleagues, it is impossible to say whether this finding is indicative of the drugs themselves or the underlying symptoms the drugs are aimed at combatting. Corticosteroids, for example, are regularly used to treat oedema and its associated neurological symptoms which can often manifest in cognitive disturbance [51, 52]. Anticonvulsants, on the other hand, are prescribed to manage seizures, and side effects can often involve disturbance in personality, cognition, or fatigue [53]; however, the limited findings warrant further investigation.

### Effect of Performance Status

The Karnofsky Performance Scale (KPS) is a scale regularly used in oncology to record the disability of a cancer patient [54]. In a mixed sample of primary and secondary brain tumour patients, Martin and colleagues investigated the relationship between KPS and CCTI performance [55]. Significant differences in performance on scales of appreciation and understanding were noted between patients with KPS scores of 90–100 and 70–80. Almost half of patients with KPS ≥ 90 and only 23% of patients with a KPS of 70–80 scored within normal range in all constructs of the CCTI. The high frequency of scores below 1.5 standard deviations from mean in at least one construct of the CCTI limits the clinical utility of using the KPS as a red flag for incapacity.

### Effect of Emotion

Across all research reviewed, age, gender, time since diagnosis, or depression did not appear to have a significant influence upon capacity. Only one paper suggested years of education and gender to have an association with capacity in brain tumour patients [29]. Mood assessment in all studies was brief and the lack of associations between depressive symptoms and incapacity is perhaps unusual, as depression is often seen to influence the information processing speed [56] and subsequent decision-making [57]. Kerrigan et al. report poor tolerance towards completing the Hospital Anxiety and Depression Scale (HADS), a relatively brief patient-reported outcome measure (PROM) [29]. Sixty-eight percent of patients who were found not to have capacity to consent to surgery using the MacCAT-T assessment were unable to complete the HADS satisfactorily. A further 13% of patients with capacity were also unable to adequately complete the test. Due to the more inclusive sampling technique used by Kerrigan and colleagues [29], such findings may be more indicative of generalised brain tumour patients’ ability to use self-report items than by other researchers in this review and explain the low influence of mood throughout the review findings. Alternatively, it is possible that mood scores were generally low across all patients and no clear association between mood and capacity could be identified. Therefore, assessment of mood should be evaluated in addition to capacity as depression is often considered a concern in regard to a patient’s capacity [58].

Suitable, simple, and clear presentation of information during shared decision-making has been proven to reduce anxiety of glioma patients prior to treatment [59]. Although simple plain language explanations should be common practice in modern medicine, a review of information needs in patients of brain tumour reveals otherwise [60]. The present review identified only one study investigating brain tumour patients’ capacity to consent to research. Due to the relatively poor outcomes of interventions available to brain tumour patients, the increasing number of clinical trials and more complex trial designs and consent forms, patients may be ‘at-risk’ of being exploited for their participation [61]. Ibrahim and colleagues report patients being fought over for their participation in clinical research [62], with coercion a possible occurrence of such research [63]. Patients may show a preconceived preference to active treatment over wait-and-see *proposals* regardless of evidence-based clinician guidance [64].

As illustrated by Kerrigan et al. the tolerability of certain PROMs is another element to consider when investigating brain tumour patients, particularly those with impaired capacity [29]. If a relatively brief and simple PROM, such as the HADS, is poorly tolerated in brain tumour patients prior to surgical intervention, more detailed and demanding PROMs are likely also heavily influenced in this population. When a patient is identified as lacking capacity during a study, researchers must make the decision as to whether or not completed PROM data are reliable. As illustrated in the present review, patients lacking capacity have the potential to make up a significant proportion of the patient population and ethically, one must consider whether continued participation in a trial is justified. Patient-proxy or observer-reported outcomes are gaining traction in clinical trials where the reliability of PROMs is brought to question.

## Conclusion

Medical decision-making capacity remains under assessed in neuro-oncology. Many of the studies trying to understand what influences capacity have focused on identifying possible cognitive links. However, cognitive performance cannot be used as a capacity test stand-in. Certain assessments, such as the SVFT, HVLT-R, and TMT A/B, may be useful in ‘flagging’ patients that may require further neuropsychological assessment. When considering the Kerrigan et al. [29] and Sullivan et al. [21] studies, it is clear that identifying those patients in need of a more detailed assessment is far more beneficial than evaluating all patients in a detailed manner. The use of ROC analysis to infer clinical utility of cognitive test performance in identifying patients who may require further assessment of capacity is more robust than correlation analysis alone. Whilst regression analyses used in Gerstenecker and colleague’s research [31••, 32••] offer statistical predictions of incapacity in brain tumour populations, the utility of this in real-world clinical settings may be more limited. The authors propose it is of greater benefit to identify rapid means of assessment than it is to present statistical models to which patient data can be imputed.

Whilst there is limited literature to suggest an influence of tumour type, grade, or location on capacity, there is a recurring theme across numerous studies suggesting tumour characteristics’ influences on cognitive abilities such as verbal fluency, memory, and executive function. Likewise, there is a limited literature to suggest any therapeutic or medicinal influences on the longitudinal effects of capacity throughout the treatment process. This is of particular importance in the brain tumour population due to the extent of treatment that is required throughout their disease trajectory. Finally, the effect of emotion on medical decision-making is poorly understood. A clinician’s duty of care should remain and serve to offer impartial advice on all treatment and research options available.

## Appendix 1. Scoping search strategy

1. Exp INFORMED CONSENT/
2. Exp Presumed Consent/
3. Exp mental Competency
4. 1 or 2 or 3
5. Neoplasms/ or exp nervous system neoplasms/
6. 4 and 5

## Appendix 2. Embase search strategy

1. exp informed consent/
2. (informed adj2 (consent or decision* or choice*)).tw.
3. informed decision making.tw.
4. informed choice.tw.
5. (consent* adj (process or form* or document*)).tw.
6. consent process.tw.
7. consent*.tw.
8. (improv* adj2 consent).tw.
9. (understanding adj2 consent).tw.
10. presumed consent.mp.
11. exp mental capacity/
12. ((competen* or capacity*) adj5 (consent* or decision* or choice*)).tw.
13. 1 or 2 or 3 or 4 or 5 or 6 or 7 or 8 or 9 or 10 or 11 or 12
14. exp central nervous system tumor/
15. ((central nervous system or CNS or brain* or cerebral* or intracerebral or intra-cerebral or intracranial or intra-cranial or spine or spinal or astrocytic or oligodendroglial or ependymal) adj5 (cancer* or tumor* or tumour* or malignan* or neoplas* or carcinoma*)).tw.
16. exp neuroepithelioma/
17. ((glioneural or neuroectodermal or embryonal or neuroepithelial or pineal or choroid plexus or teratoid or rhabdoid) adj5 (tumor* or tumour*)).tw.
18. (glioma* or glial* or astrocytoma* or xanthoastrocytoma* or glioblastoma* or gliosarcoma* or oligodendrogli* or oligoastrocyt* or ependym* or subependym* or astroblastoma* or ganglioglioma* or gangliocytoma* or neurocytoma* or liponeurocytoma* or pineocytoma* or pineoblastoma* or medulloblastoma* or neuroblastoma* or ganglioneuroblastoma*or medulloepithelioma* or GBM*).tw.
19. 14 or 15 or 16 or 17 or 18
20. 13 and 19
21. limit 20 to embase
